# Coping with interoperability in the development of a federated research infrastructure: achievements, challenges and recommendations from the JA-InfAct

**DOI:** 10.1186/s13690-021-00731-z

**Published:** 2021-12-09

**Authors:** Juan González-García, Francisco Estupiñán-Romero, Carlos Tellería-Orriols, Javier González-Galindo, Luigi Palmieri, Andrea Faragalli, Ivan Pristās, Jakov Vuković, Janis Misinš, Irisa Zile, Enrique Bernal-Delgado, on behalf of the InfAct Joint Action consortium, Brigid Unim, Flavia Carle, Rosaria Gesuita, Damir Ivanković, Marko Brkić, Jelena Dimnjaković, Jane Lyons, Ronan Lyons, Zeynep Ors, Metka Zaletel, Paulo Nogueira, Luís Velez Lapão, Håkon Haaheim, Petronille Bogaert, Linda Abboud, Herman van Oyen

**Affiliations:** 1 Biocomputing Unit, Institute for Health Sciences in Aragon (IACS), Zaragoza, Spain; 2 Data Sciences for Health Services and Policy Research, Institute for Health Sciences in Aragon (IACS), Zaragoza, Spain; 3 Department of Cardiovascular, Endocrine-metabolic Diseases and Aging, Istituto Superiore di Sanità-ISS, Rome, Italy; 4 Center of Epidemiology and Biostatistics, Polytechnic University of Marche, Ancona, Italy; 5 Institute of Public Health, Zagreb, Croatia; 6 Centre for Disease Prevention and Control, Riga, Latvia

**Keywords:** Health data, Secondary use of data, Legal interoperability, Organizational interoperability, Semantic interoperability, Technological interoperability, Federated research infrastructure, Distributed solutions

## Abstract

**Background:**

Information for Action! is a Joint Action (JA-InfAct) on Health Information promoted by the EU Member States and funded by the European Commission within the Third EU Health Programme (2014–2020) to create and develop solid sustainable infrastructure on EU health information. The main objective of this the JA-InfAct is to build an EU health information system infrastructure and strengthen its core elements by a) establishing a sustainable research infrastructure to support population health and health system performance assessment, b) enhancing the European health information and knowledge bases, as well as health information research capacities to reduce health information inequalities, and c) supporting health information interoperability and innovative health information tools and data sources.

**Methods:**

Following a federated analysis approach, JA-InfAct developed an ad hoc federated infrastructure based on distributing a well-defined process-mining analysis methodology to be deployed at each participating partners’ systems to reproduce the analysis and pool the aggregated results from the analyses. To overcome the legal interoperability issues on international data sharing, data linkage and management, partners (EU regions) participating in the case studies worked coordinately to query their real-world healthcare data sources complying with a common data model, executed the process-mining analysis pipeline on their premises, and shared the results enabling international comparison and the identification of best practices on stroke care.

**Results:**

The ad hoc federated infrastructure was designed and built upon open source technologies, providing partners with the capacity to exploit their data and generate dashboards exploring the stroke care pathways. These dashboards can be shared among the participating partners or to a coordination hub without legal issues, enabling the comparative evaluation of the caregiving activities for acute stroke across regions.

Nonetheless, the approach is not free of a number of challenges that have been solved, and new challenges that should be addressed in the eventual case of scaling up. For that eventual case, 12 recommendations considering the different layers of interoperability have been provided.

**Conclusion:**

The proposed approach, when successfully deployed as a federated analysis infrastructure, such as the one developed within the JA-InfAct, can concisely tackle all levels of the interoperability requirements from organisational to technical interoperability, supported by the close collaboration of the partners participating in the study. Any proposal for extension, should require further thinking on how to deal with new challenges on interoperability.

## Introduction

“Information for Action!” is a Joint Action (JA-InfAct) on Health Information promoted by the EU Member States and funded by the European Commission within the Third EU Health Programme (2014–2020), including 40 partners in 28 EU and associated countries. The main aim of the JA-InfAct is to build an EU health information system infrastructure and strengthen its core elements by a) establishing a sustainable research infrastructure to support population health and health system performance assessment, b) enhancing the European health information and knowledge bases, as well as health information research capacities to reduce health information inequalities, and c) supporting health information interoperability and innovative health information tools and data sources. One of the underlying tasks has been setting up the pillars for the design, preparation and implementation of a federated research infrastructure (FRI) that leverages the use of health data to carry out policy-oriented research.

**Fig. 1:**
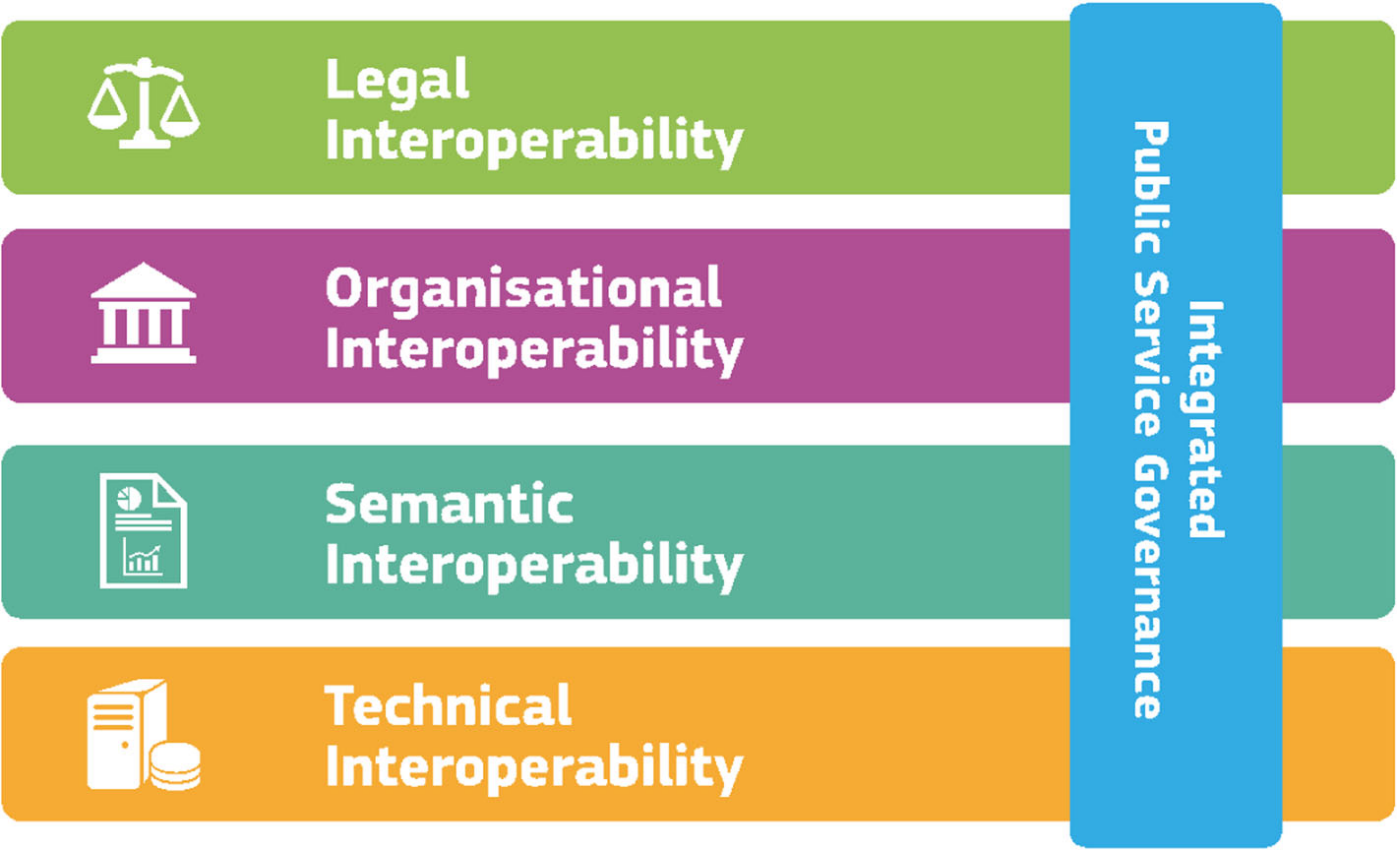
European Interoperability Framework interoperability model

Paramount in the development of a federated research infrastructure, where data is leveraged from multiple and heterogeneous data sources hosted in multiple sites with different governance models, is interoperability. JA-InfAct interoperability elements are framed in the recommendations report by the European Interoperability Framework (EIF) [1]. According to the EIF, interoperability is defined as *“﻿the ability of organisations to interact towards mutually beneficial goals, involving the sharing of information and knowledge between these organisations, through the business processes they support, by means of the exchange of data between their ICT systems*”. In the specific case of JA-InfAct, interoperability refers to the capacity to capture coherent data in the different partners, being able to reproduce the same analyses and being capable of sharing the results of these analyses. The design, development and implementation of the JA-InfAct federated research infrastructure have built upon the interoperability layers introduced by the EIF and depicted in Fig. 1. The EIF framework classifies the interoperability elements in four layers that need to be addressed for a successful interoperable (public) service -namely Legal, Organisational, Semantic and Technical interoperability.

This paper seeks to describe the process and challenges followed in the context of JA-InfAct to cope with the different layers of interoperability when trying to answer population health research queries in the context of a federated infrastructure. Likewise, the report provides recommendations for the eventual real-life implementation of such a federated infrastructure.

## Methods

JA-InfAct has addressed the different interoperability challenges to build an FRI, following an approach based on case studies (also known as pilots). In Table 1, there is a synthesis of the three use cases deployed along the Joint Action. The selection had mainly to do with different levels of maturity; from the simplest definition of a study and how it materializes into the required documentation (i.e., case study on dementia), the definition of a population based indicator that materializes in a SQL query that is distributed to elaborate indicators in-house (i.e., case study on resilient populations), to a completely distributed exercise where a common data model is built, an interoperable analytical pipeline is developed and distributed among a number of nodes that run the analyses and produce the research outputs, that are sent back to a Coordination Hub that summarises the results. Ultimately, this case study is complex enough to provide general lessons on how to cope with the different layers of interoperability when building a federated approach as it covers critical issues in terms of: a) data requirements (linkage of individual sensitive data from multiple data sources that have to be pseudonymised at origin); b) study design requirements (development of cohorts including thousands of individuals with multiple exposures, followed over time); c) semantic and syntactic interoperability requirements (building a complex common data model across different encoding systems); and, d) harmonization of technological interoperability across a variety of settings with uneven capabilities (implementing and deploying full distribution algorithms embedded in a docker solution). (*n.b.* a more extensive description of the three use cases is at https://www.inf-act.eu/wp10).

**Table 1:** JA-InfAct use cases

Use case	Purpose	Data sources	Common Data model (main entities)	Distribution	Hubs
Dementia care	Identification of 1-year follow up contacts and associated costs	Insurance data PC EHR Hospital stays Prescriptions ER data RHB contacts Billing data	Individual patient Care provider Point of care	Data model specification (v0.1)	Aragon (ES) France (FR)
Desirable health services utilization	Elaboration of a population-based health indicator based on those lower users	Insurance data PC EHR Prescriptions Hospital stays	Individual Insurees Residence	Protocol, data model specification and SQL script for data transformation (v1.0)	Wales NHS (UK) Aragon (ES)
Stroke care pathway	Discovery of the actual care pathway for Acute Stroke patients	Insurance data ER data Hospital data	Individual patient Care provider Residence/Healthcare area Episodes Events	Complete solution: Docker with open source Log builder and Process Mining FAIR publication (v14.0)	Aragon (ES) Marche (IT) HU Zagreb (HR) HU Riga (LV)

The last approach is the one that has defined the actual FRI solution, providing full insight on how to make interoperability a reality. In short, the JA-InfAct federated analysis infrastructure is an ad hoc infrastructure solution proposed to perform a cross-border analysis, where different infrastructures, information models, and even legal frameworks may apply. The core element of the analysis infrastructure is the process-mining methodology. Process mining is a series of data analysis techniques able to discover, analyze, and improve business processes based on event data. In this context, process mining is applied to real-world datasets that are combined and processed to generate the care pathways process models within the premises of each participant partner.

The analysis methodology is encapsulated in a software distribution solution that acts as the central element of the federated analysis infrastructure, that leverages the data capture and execution of the analyses across different partners and the exchange of the results. All the methodologies and solutions are designed and implemented following *security- and privacy-by-design* approaches and to fulfil the interoperability challenges.

This section is organised to describe these three elements, using as a case study the empirical discovery of care pathways of acute ischemic stroke. Subsection 2.1 summarises the process-mining analysis, further commented in [2]. Subsection 2.2 details the technical aspects of the federated analysis infrastructure, considering the software elements and the orchestration elements to reach the desired solutions. Subsection 2.3 focuses on all the interoperability elements that have been considered within the first two elements (the analysis methodology and the analysis infrastructure) and specific organisational agreements reached between partners for a successful deployment of such an infrastructure.

### Description of the stroke use case: a process-mining based analysis methodology

For the purpose of the stroke use case, the process-mining methodology used in the JA-InfAct was the one previously introduced for a similar use case at a regional level in the work by González-García et al. [2]. It is based on analysing real-world datasets related to stroke care (urgent care episodes, hospitalisations and patient socioeconomic data and among others) using process mining techniques [3, 4], and, specifically, process discovery. Process discovery techniques aim to infer the process model of a business process by analysing the event logs, i.e. the registry of time stamped activities that compound the specific business process of interest. In the use case setting, the event logs used are derived from the real-world datasets mentioned and the resulting process model is then the empirical stroke care pathway. The final objective of the analysis methodology is then to detect how this empirical pathway differs from theoretical pathways of care or clinical guidelines, such as the acute ischemic stroke care pathway, namely the *Code Stroke*, defined in [5].

**Fig. 2:**
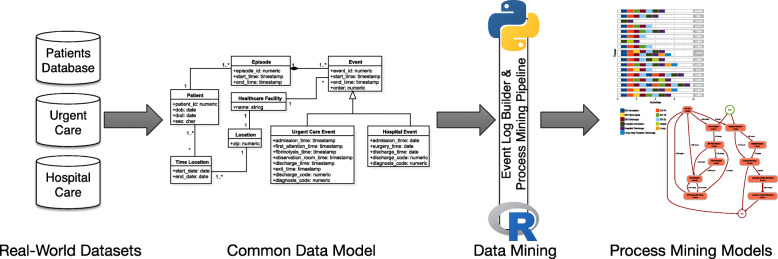
Methodology and analytical pipeline supporting the case study on stroke

The methodology is illustrated in Fig. 2 and comprises four main elements: 1) capture the real-world data to be further processed, considering that starting of the acute stroke care pathway should be captured in the urgent care (i.e. accident and emergency care services) and that hospital care information systems and patients’ information database should contain specific details about patients characteristics; 2) transform the data from their specific information systems into a defined common data model, which contains the actual semantics of the contents in the form of different entities (i.e., patients, visits, procedures, etc.), the variables that define these entities (i.e., age or sex for patients, visit date and hospital, or procedure date and code), their relationships (i.e., when a patient visited a hospital where he or she received a procedure) along with the translation from the encoding systems prevalent in the different nodes (ie, ICD 9th, ICD 10th, SNOMED, etc) to a common one; 3) process the data stored in the common data model to generate an event log (where each register in the data set represents an activity and its attributes) using the *Event Log Builder* tool (the piece of software that translates the real-world datasets into actual event logs) that feeds the final *Process Mining Pipeline* tool, which generates the empirical process models; and 4) compare and contrast the process models obtained to verify the actual care pathways in the different countries. The outputs of the methodology are the process models that can be depicted, for example, as process maps that present the real-life transitions between events, the number of patients along each trajectory and throughput times (see later in the results section).

### Description of the federated infrastructure

The JA-InfAct federated analysis infrastructure has been designed with the objective of distributing the analytical pipelines using the data of the different partners, in this case study replicating the process mining methodology without requiring any individual data movement from one partner to another, or to a *Coordination Hub*. The Coordination Hub, in this context, is responsible for developing the analysis scripts, supporting each participant partner in their deployment and producing insights from the comparison between partners’ outputs. The term *federated infrastructure* is used because participating partners can act independently without requiring the rest of the partners to perform the analyses.

**Fig. 3:**
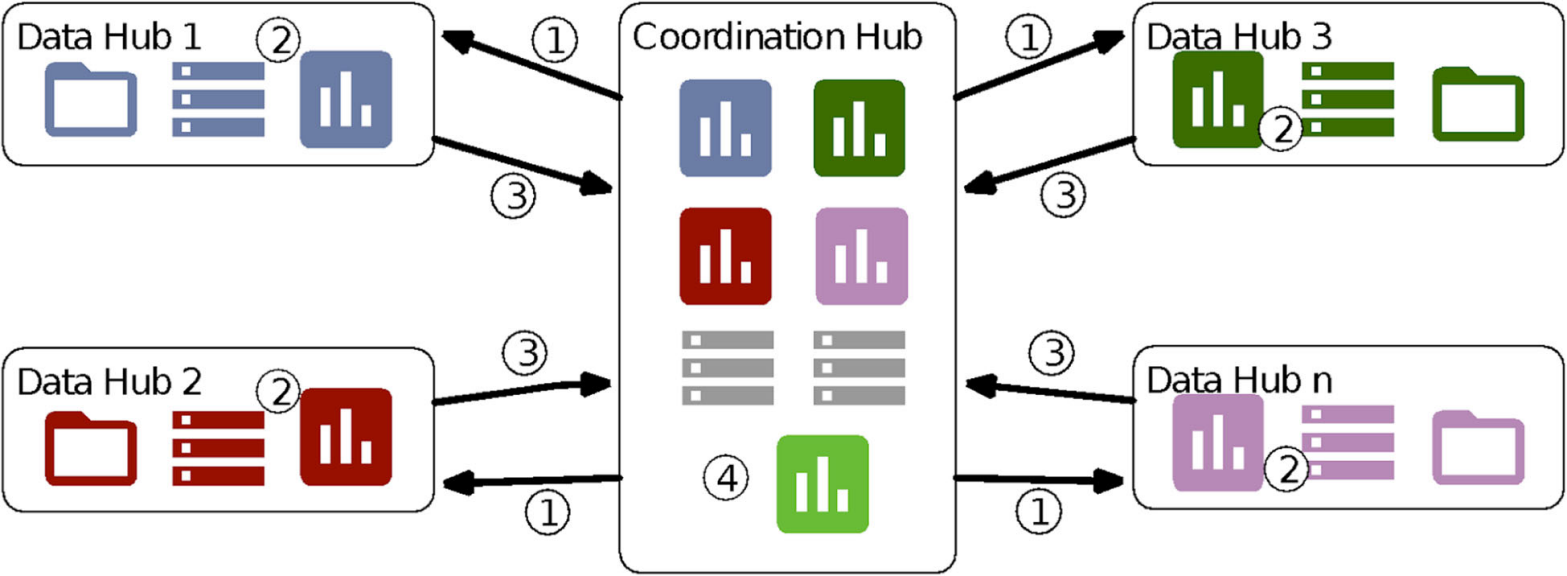
JA-InfAct federated analysis infrastructure

Figure 3 presents the schema of the distribution workflow. The partners involved in the infrastructure are generally considered *Data Hubs* in the Figure. They are responsible for transforming and loading their own data sets in the common data model format previously defined and agreed on. As stated initially, this is an ad hoc infrastructure, which means that the code distribution is actually done in two steps: first, the Coordination Hub encapsulates the process mining analysis scripts into a portable execution environment and, second, each Data Hub gets this portable execution environment, deploys it and runs it in their premises, indicating where the data is located.

The analysis process starts, point 1 in the Figure, when the Coordination Hub distributes the process mining analysis scripts among the Data Hubs. Next, in point 2 of the Figure, partners fetch the scripts into their systems, indicate the input data placement to the analysis code and run the analyses. As a result of this point, each partner obtains their own results (ie, stroke care pathways). Finally, depicted as point 3 of the Figure, partners send back their results to the Coordination Hub. This feedback in the form of local results is sent in this case manually, by compressing the output dashboard and mailing it to a specific address. Finally, once the Coordination Hub has gathered information from all partners, it compares them to present a final analysis, point 4 in the Figure.

Note that this architecture follows the *privacy-by-design* principle as all data is governed under the legal provisions of the hosting institutions and no individual data is moved outside partner premises tackling various relevant legal interoperability issues raising legal barriers to cross-border data sharing. Results and data that feeds back from partners to the Coordination Hub are always outputs, in this case, aggregated measures in the form of the process models, and runtime application error logs. In addition, following the *secure-by-design* principle and as a way to build trust among federated infrastructure participants, it is important to remark that all the analysis and deployment solutions presented are open source, thus all source code of the scripts for data management and analysis are auditable by the participants, ensuring the credibility on what is going to be executed, enhancing the reliability of the results and helping to increase the solution quality through the contribution from the partners.

### Interoperability layers

What has been described in the two previous subsections relies on guaranteeing the interoperability among the different components of the solution, as well as the relationship within the involved partners in terms of trust, governance and legal compliance. In the following subsections we describe how the different layers of the EIF have been achieved in the context of the JA-InfAct federated research infrastructure.

#### Legal interoperability. The general data protection regulation

The top layer in the EIF interoperability model is legal interoperability. Given the compliance with GDPR and overarching Ethical principles, legal interoperability is about “*ensuring that organisations operating under different legal frameworks, policies and strategies are able to work together*”.

Being the objective of this federated infrastructure to analyse care pathways using patients’ data, the legal framework to be considered are those regulating the use of personal health data for research purposes. There has been a huge effort by European legislators in this topic so as to homogenize the use of this kind of data across the EU Member States, materialised in the General Data Protection Regulation (GDPR) [6], that is therefore mandatory for the JA-InfAct federated infrastructure. In the development of the federated infrastructure, a vast effort has been put on guaranteeing the GDPR compliance, mostly preserving all personal data in the premises of the data controllers (i.e., in the partners information systems), and interchanging only aggregated information with the Coordinator Hub for its further meta-analysis and publication, what could be understood as a differential privacy approach.

#### Organisational interoperability. The JA-InfAct proposal and grant agreement

The second layer to be discussed is the organisational interoperability layer. Organisational interoperability refers “﻿*to the way in which public administrations align their business processes, responsibilities and expectations to achieve commonly agreed and mutually beneficial goals*”.

The JA-InfAct federated analysis infrastructure was designed as a client-server infrastructure, with a Coordination Hub orchestrating the whole process (consolidation of the data model, development of the analytical pipeline including technological solutions) and a node counterpart where a contact person performed the following duties: 1) detect the staff with the knowledge to perform the required tasks; 2) assist to the work meetings to ensure the proper coordination of the work; 3) commit the necessary resources to develop the work; 4) provide the required feedback to improve and solve the possible issues that appear during the development; and 5) carry on the analyses and feedback and interpret the results considering the local context.

#### Semantic interoperability. The common data model and the data codifications

The semantic interoperability ensures that “*the precise format and meaning of exchanged data and information is preserved and understood throughout exchanges between parties*”. So, in practice, it is required to guarantee that, when performing the care pathway analyses in the different partners, the data and the results refer to the very same caregiving processes.

The cornerstone element in the semantic interoperability layer is the common data model (CDM), in which the data entities and their relationships are defined, and serves as the common storage to perform further analyses. The CDM design was iteratively refined to express the actual caregiving settings in the different partners. For example, initial versions of the fibrinolysis treatment and the thrombectomy procedure, key for a rapid response on ischaemic strokes, were not considered to appear within urgent care events, while in further refinements these activities were included in this setting. At the moment of writing this paper, the CDM was on its 14th revision.

It is important to note that, as a fundamental component of the CDM, apart from the entities and relationships, the data model specified the encoding systems or standards to be used when codifying the model variables in the different nodes of the federation.

#### Technical interoperability. The event log build and the process mining pipeline

The technical interoperability layer covers *“﻿the applications and infrastructures linking systems and services. Aspects of technical interoperability include interface specifications, interconnection services, data integration services, data presentation and exchange, and secure communication protocols*”.

The most important element in the technical interoperability layer of the JA-InfAct federated analysis infrastructure is the “*package deployment system*”, i.e., how the different source code of the analysis scripts is encapsulated so as to be easily transmitted from the Coordinator Hub to the partners, easily executed in the partners to perform the analysis in their premises and, the results, easily transmittable back to the Coordination Hub.

As a final point regarding the technical interoperability, it is important to highlight that the analysis code distributed to the partners relies on having the input data of each partner in the CDM format. It is the responsibility of each partner to create the extraction, transformation and load (ETL) processes that capture the required data from their health information systems and fill the CDM according to the agreed definitions.

## Results

The JA-InfAct federated infrastructure, as designed, has been able to yield the intended output in the four nodes composing the case study on stroke -each node has been able to produce a dashboard depicting the real-life care pathways in the four countries. This section details the actual implementation of the process mining methodology shown in Fig. 1 within the JA-InfAct FRI. Therefore, it includes the specification of the common data model, distribution of the analytical pipeline, implementation of the pipeline in the different nodes and the collection of the outputs.

### Specification of the data model

The research question sent out to the federation materialized in: 1) a definition of the cohort of patients (inclusion criteria) as those who, in the period of study, had been admitted with symptoms of stroke in an emergency ward or had a hospital admission due to stroke; 2) because of the different theoretical pathways, a classification of patients as confirmed ischemic stroke or hemorrhagic stroke patients; and, 3) the definition of the different activities (namely, events) that patients would follow in their journey from admission to discharge (Table 2).

**Table 2:** Types of activities considered in the process mining analysis

Activity Name	Activity Description
**ER Admission**	Administrative admission to Emergency Room Department
**ER First Attention**	First contact with an MD in the ER Department
**ER CT**	Computed Tomography Scan imaging at ER Department
**ER Fibrinolysis**	Fibrinolysis infusion at ER Department
**ER Thrombectomy**	Thrombectomy surgical procedure at ER Department
**ER Observation Room**	Observation room stay at ER Department
**ER Discharge**	ER Department administrative discharge
**ER Exit**	ER Department physical exit
**Hospital Admission**	Hospital administrative admission
**Hospital Fibrinolysis**	Fibrinolysis infusion during hospitalisation
**Hospital Thrombectomy**	Thrombectomy surgical procedure during hospitalisation
**Hospital Discharge**	Hospital administrative discharge
**Long-stay Hospital Admission**	Long-stay (recovery) hospital administrative admission
**Long-stay Hospital Discharge**	Long-stay (recovery) hospital administrative discharge

As a second step, coding experts and neurologists among different partners were consulted to select the codes that conceptually better fitted the cohort definition. In our case, and for the nodes included in the exercise, we just needed to map between ICD-9th [7] and ICD-10th [8] codes.

Once the cohort of patients was defined and the potential activities were identified and discussed, the data model was iteratively refined to express the actual caregiving settings in the different partners, so as to get a CDM. For example, initial versions of the fibrinolysis treatment and the thrombectomy procedure, key for a rapid response on ischaemic strokes, were not considered to appear within urgent care events, while in further refinements these activities were included in this setting. At the moment of writing this paper, the CDM was on its 14th revision.

**Fig. 4:**
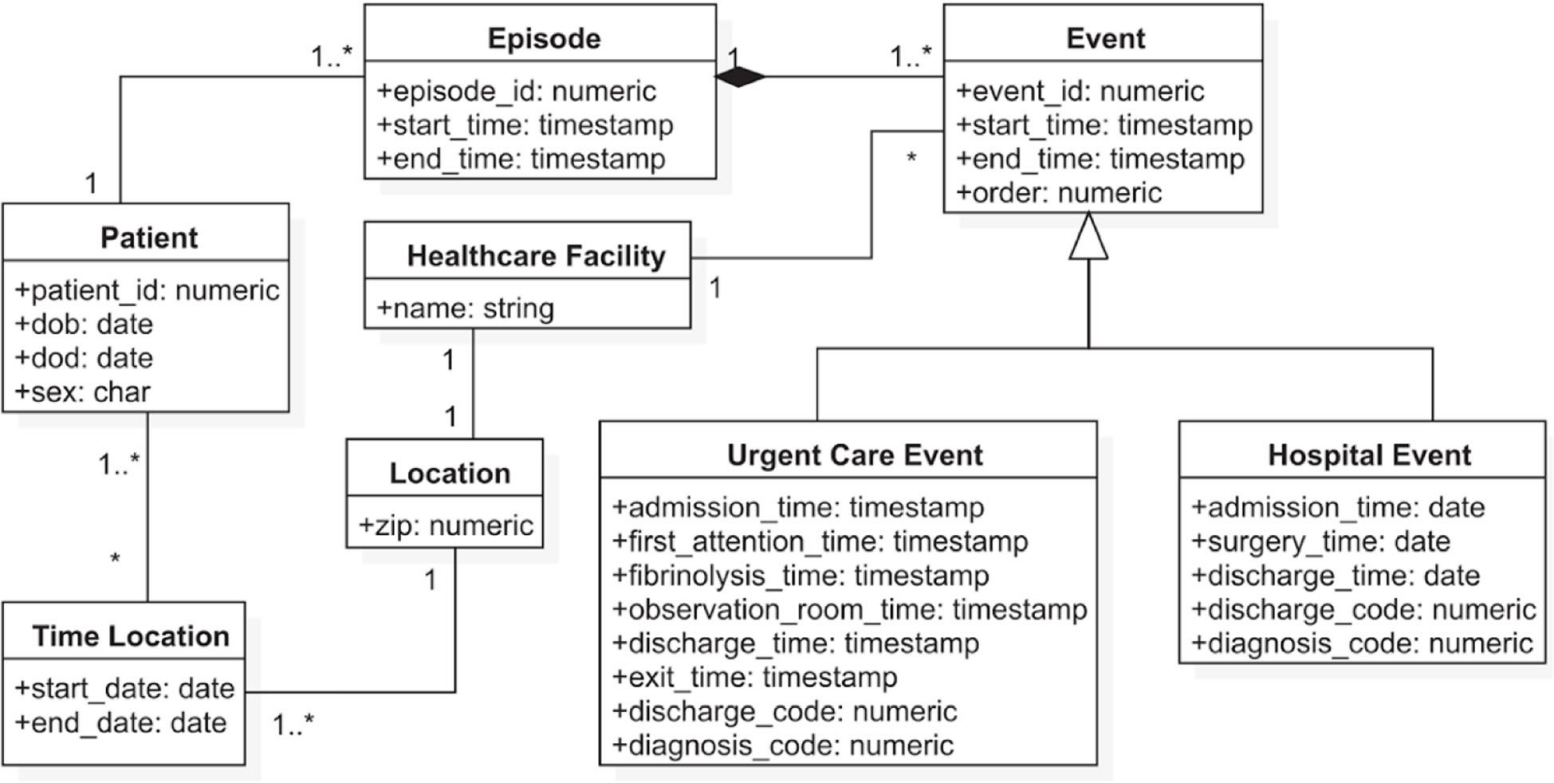
Stroke care data model - Conceptual diagram

As a final result, the common data model that supported the development of this case study, comprised five main entities: patient, care provider, residence/healthcare area, episodes and events (activities). The data model is shown in Fig. 4 using the Unified Model Language^1^ conceptual model notation.

The CDM for this case study can be found here 10.5281/zenodo.4879504

### Analytical pipeline

Once the data model was common to all the nodes in the federation, the institution in charge of the Coordination Hub (IACS) developed and distributed the analytical pipeline; in this case, distributing two main pieces: a) the Event Log Builder, in the form of a Python script; and, b) the Process Mining Pipeline script, written in the R language.

Both pieces of software were packaged using Docker [9]. Docker is a compendium of existing Linux-based technologies that, in simple terms, is able to create isolated execution environments where software developers can guarantee that: 1) will be exactly the same wherever they are executed; 2) code dependencies are easily managed; and 3) the deployment of the execution environments in different locations (in the current, case, the different partner premises) is transparently managed. The first point is provided by using operating system containerization; the second point is provided by having a (nearly) infinite set packages for existing code libraries, and the third point is provided by having an environment exchange hub solution, publicly under namely the Docker Hub or privately deployed by software developers on their premises, where the execution environments are uploaded by their authors and easily downloaded by the users. For the purposes of this case study we adapted the Docker solution used in another project, available at https://hub.docker.com/repository/docker/iacsbiocomputing/ictusnet_analysis.

#### Implementation of the pipeline and production of research outputs

The main tangible outcomes of the JA-InfAct federated infrastructure are a number of interactive dashboards generated on each partner premises on the actual care pathways followed by patients with a suspicious stroke in Marche (IT), Riga (LV), Zagreb (HR) and Aragon (ES).

**Fig. 5:**
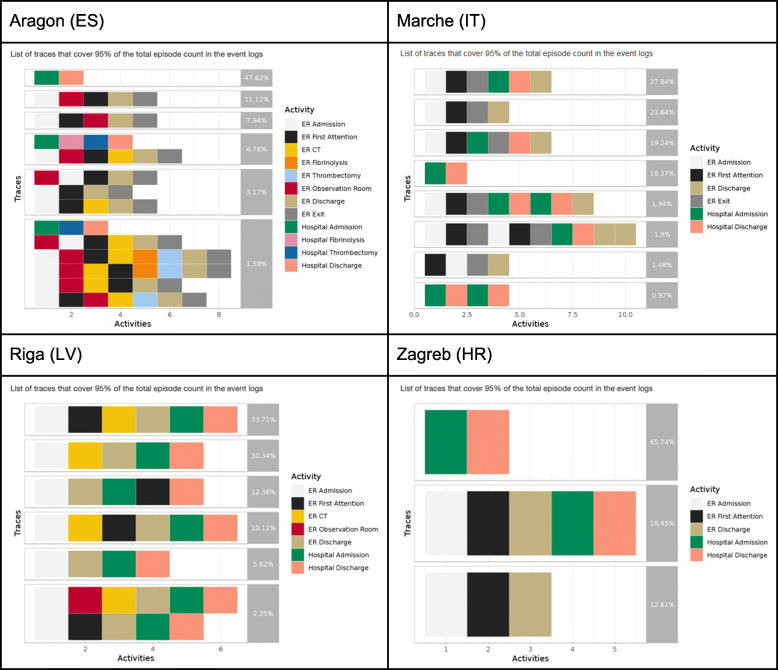
Process traces

**Fig. 6:**
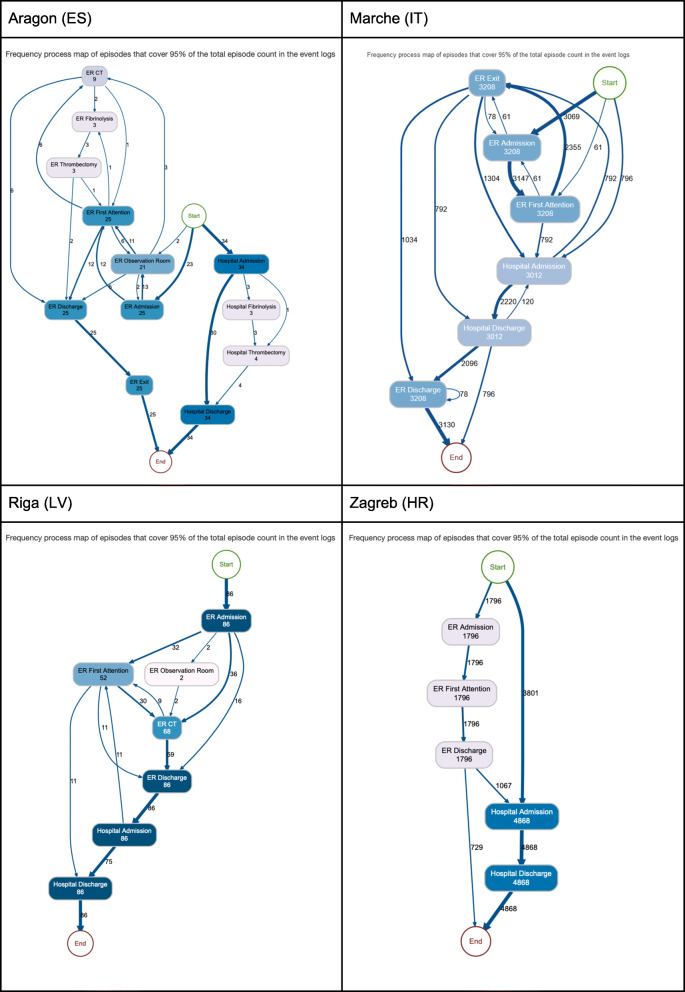
Number of patients across the pathway

**Fig. 7:**
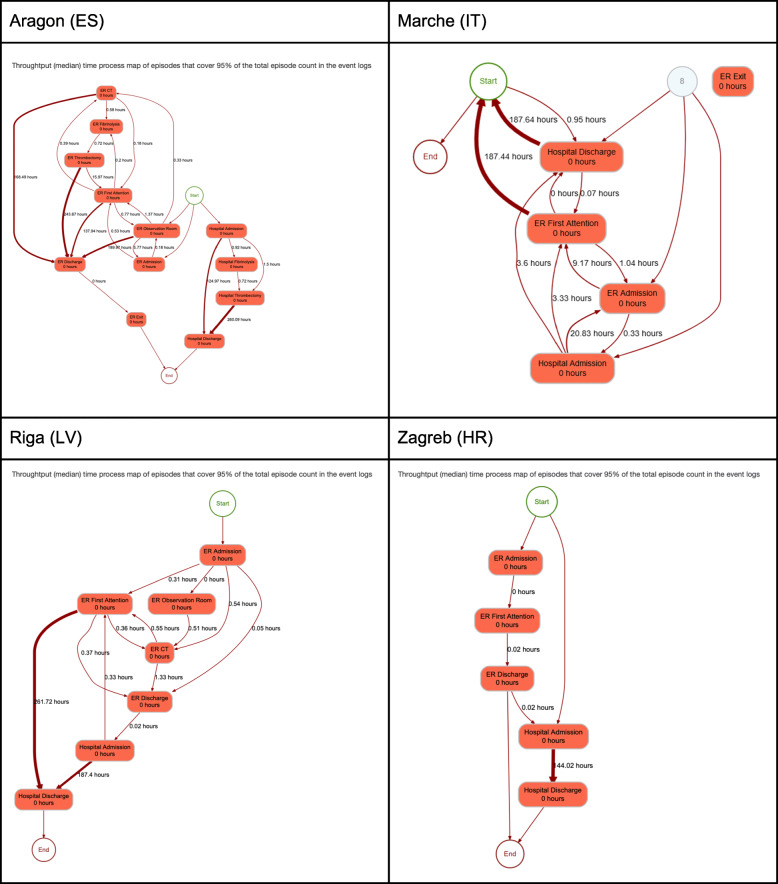
Throughput times

**Fig. 8:**
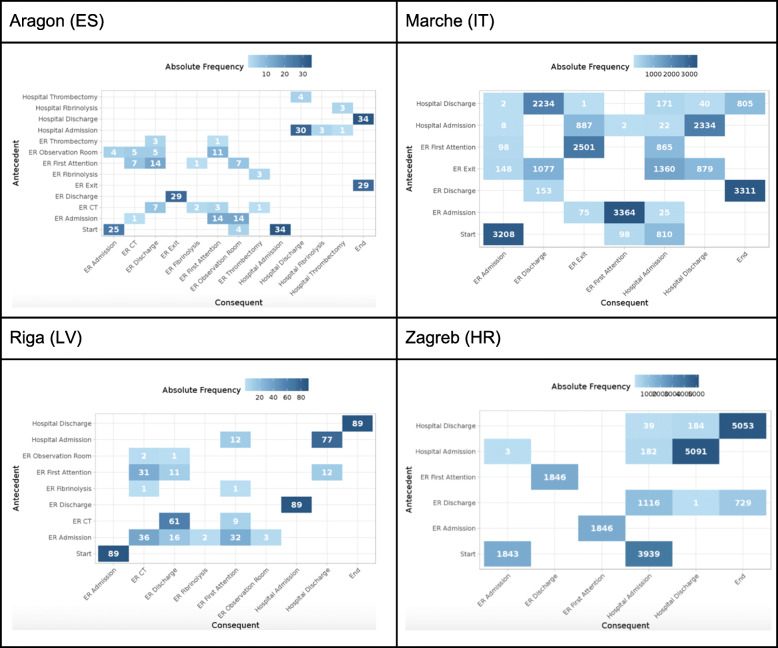
Matrix of precedence

The interactive dashboards contain the sequence of activities followed by the patients in the cohort in each of the sites (namely, process traces in Fig. 5). Next, it contains the process map with the amount of patients moving throughout the care pathway (Fig. 6) and the throughput times among activities (Fig. 7); and, finally, it also contains the precedence matrix, a presentation of the information present in the process map with frequency information (frequency of transitions between caregiving activities analyses) in a matrix form (Fig. 8).

Once the different nodes have been able to implement the analytical pipeline and produce the aforementioned outputs, dashboards are sent back to the Coordination Hub for further analyses. Thanks to the effort done in the interoperability elements the comparability of the dashboards was guaranteed.

The different dashboards, with outputs for all patients, ischaemic strokes and hemorrhagic strokes can be consulted here 10.5281/zenodo.4925733 (*n.b*. It is important to stress that the process maps, process traces and precedence matrices are purely aggregated information, so no personal data is given. In addition, only those processes that sum the 95% of the total episodes analysed are published, avoiding a rare re-identification possibility of having a single patient following a singular caregiving process).

## Discussion

Having completed the exercise we have demonstrated the feasibility of the JA-InfAct approach to federated analyses. All the layers of interoperability have been successfully considered in this case study. The exercise has provided meaningful insight into the difficulties of the implementation of such a federated infrastructure and has shed light on the ways forward. In the following section, we specifically address the challenges faced, also providing recommendations on how to tackle them in the eventual development of a federated research infrastructure.

### Challenges on legal interoperability

In the JA-InfAct federated analysis infrastructure, the GDPR compliance has been materialized avoiding the use of personal data during the analysis, minimising the data requirements to complete the case study and providing reliable procedures on how to manage the data at the participant partner’s premises within their governance and security assurance procedures. All these concerns are captured in the common data model and how it is filled and processed. The common data model and the data processing designed in [2] had the following characteristics to ensure GDPR compliance, especially those related to Art.5, related to personal data processing and Art.89, which regulates the research uses of personal data: 1) the data has been only used for the agreed analyses between partners (‘*purpose limitation’*); 2) the data gathered has been limited to only those variables required for the analyses and the analysis time-frame (‘*data minimisation*’); 3) the personal data has been stored using pseudonyms to avoid possible reidentification of patients (‘*confidentiality’*), and 4) patients’ data is only stored on the premises of the partners which has the mandate/responsibility of curating such original datasets (‘*storage limitation*’).

### Challenges on organisational interoperability

Organisational Interoperability is rooted in the willingness of the Data Hubs to participate and cooperatively respond to a research question that is relevant to the participants. It is labour-intensive and requires almost continuous communication between all the partners and the Coordination Hub and building a trust relationship based on the adjustment of use case specifications considering all inputs from participant partners, reaching consensus on each step of the process, allocating task to staff with relevant skills in each organisation through the different steps in the process and being completely transparent in all developments aimed at producing a full reproducible implementation of the analytical pipeline.

The JA-InfAct federated infrastructure has been designed as a client-server infrastructure with a Coordination Hub that orchestrates and supervises the whole process in a quite controlled modest case study. Key elements in terms of organisational interoperability have been to get a common understanding of the procedures and the roles of each of the parties, including data access; and facing the challenges regarding the technical deployment of the infrastructure.

As for the first, it has been important to take the time to clarify the roles of the different actors in the federation. So, it has been needed to extensively explain the Coordination Hub, as the one orchestrating all the procedure from the development of the data model, the implementation of the analytical pipeline, the adaptation of the technological solutions to the computational environment of each node, acting as a *help desk* with the contact persons in each node, and the supervision of the process so as to synchronize the work in the different nodes. Most importantly, it has been necessary to explain the roles and requirements for the nodes as those that were basically in charge of providing information on the actual data access and ability to comply with the common data model and provide insight to adapt it to local specificities, effectively access to the required data, and above all set up a team in house that is able to comply with the requirements of the case study, particularly the deployment of the technological solutions.

As for data access challenges, the case study was designed to get the intended outputs with a very simple data model and rather limited data requirements. So, in this very controlled context, questions such as linkability of data sources, insufficient coverage or lack of relevance of the data sources, or more in depth data quality elements at variable level such as incompleteness, missingness or systematic errors have not been deemed as big challenges to deal with in this very exercise.

So, the main challenge in the deployment of the federation was expected to be (and it has been) the need for technical capacities in some of the nodes of the infrastructure, in particular, IT profiles that can easily implement or deploy the technological solutions developed by the Coordination Hub. An extra effort on supervision and capacity building has been assumed by the Coordination Hub that could not be sustainable in the eventual scaling up of the infrastructure.

### Challenges on semantic interoperability

As in any cross-national comparative research, semantic (and syntactic) interoperability is the main challenge. Each of the entities composing the data model (patient, contact, event, time) are subject to threats to semantic (and syntactic) interoperability. As a matter of example, the definition of the cohort (how to define stroke and refine the strategy to select stroke patients); how specific or sensitive is the definition of ischemic or hemorrhagic stroke, the definition of episode, the identification of the care activities and where these activities are provided; and if these activities can be separate out across care providers, the uneven granularity of the timestamps, or how exiting the process is defined.

The effort made to get the data model common has implied understanding the care processes in the different nodes of the federation, agreeing on common concepts (and then, definitions) for the different attributes within entities, if there were different standards or encoding systems, building the appropriate cross-walks, transforming the variables when needed to a common format or, in the worst scenario, reaching a minimum common denominator.

In this very exercise, the main threats to semantic (and syntactic) interoperability that the Coordination Hub had to solve have been:

- Reaching a consensus on the specification of the relevant activities to map in terms of acknowledging the hyper-acute care process in stroke (i.e. relevant therapies such as fibrinolysis or mechanical thrombectomy, but also CT image, etc.)
- Reaching a consensus on the classification of a stroke as ischaemic, transient ischemic or hemorrhagic using both ICD-9th and ICD-10th.
- Defining normalized dictionaries for certain concepts, such as ‘discharge_code’, based on mapping all existing categories in each partner with similar descriptors.
- Setting a common default timestamp granularity and establishing the rules to comply with such granularity while being consistent with the care process, even when such level of granularity was not available at each site (i.e., requiring date-time granularity with 00:00:00 time when only date was available and checking the consistency of the timestamps on an expected sequence of activities to assess irregularities).
- Establishing a normalized file format (CSV file, comma separated, without quotation marks) and encoding (UTF-8) with preset headers and fixed variable name fitting the common data model specifications.

### Challenges on technological interoperability

As stated previously, so as to guarantee full technical interoperability, the analysis scripts should be able to run independently of the available systems in the partners with all dependencies included within the containerized solution. Then Docker-based deployment relies on the availability of operating system level support for Docker containers among partners, commonly Linux-based servers.

However, some partners do not have Linux servers. For those cases the Coordination Hub implemented a set of virtual machine images containing Linux plus the Docker software, replicating the native environment previously described. These images were created in Open Virtualization Format version 2 [10] and Virtual Hard Drive version 2 format [11]; and can be deployed in virtually all commercial systems (Microsoft Windows, Apple macOS, multiple *NIX variants, etc.) and have demonstrated their utility during the project development. For the purposes of this case study the images used were adapted from the virtual machine images used in [12].

### Upcoming challenges and recommendations

#### On legal interoperability

In the real life implementation of a federated approach, where many more nodes with different responsibility on the data curation and management, many more data sources could be used and data requirements could be larger in a context where research questions and queries grow exponentially. In this case, assuring the compliance with GDPR principles, in particular minimization and confidentiality, gains relevance and imposes new actions. In this context, Data Protection Officers (DPOs) will play a major role.

##### Recommendation 1

In the real life expansion of the JA Infact, data access will require to document how GDPR principles will be assured, mainly throughout: a) a protocol of the study behind the query (including the purpose and methodology) and a data management plan including the data schema (entities, variables, operational description with categories and values, and encoding systems), what are the measures to assure confidentiality and minimisation; who will be the actors and what role they will pay in data management and for how long.

##### Recommendation 2

In the context of the nodes, there will be a need for DPOs to understand how data accessing and data management procedures will work in the context of a federated approach. Specific training programs for DPOs could be recommendable. The other way round, the continuous exchange with DPOs will make each node be aware of the local and specific requirements and anticipate the data accessing needs.

##### Recommendation 3

In a scaled context, there will be a need for technological solutions that ensures *security-* and *privacy-by-design*. Robust authentication and authorization systems will be needed to manage data access to only authorized users and to provide traceability information for forensic analyses in the follow up of a given user.

#### On organisational interoperability

In the JA-InfAct case study the number of actors interacting has been confined to a few: in the Coordination Hub a technological and a domain expert, in the different nodes one or two contact persons with mixed profiles. In this very controlled case study, bilateral interaction between the Coordination Hub and the four participan nodes, close tutoring of the process, even online remote intervention can be used to solve queries on the deployment of the technological solutions.

As mentioned when describing the federated research infrastructure, a good wealth of tasks are developed inhouse by each of nodes within the federation - discussing of the research question, agreeing on a common data model, accessing and collecting the data in the way required by such a data model, deploying the technologies developed elsewhere in their technological infrastructures, running the scripts, interpreting the error logs and the outputs.

In a federation with much more nodes, or in a hybrid federation with one node serving as an orchestrator of other nodes, or in a peer to peer federation where any node can orchestrate or any node can interrogate the federation the needs for organisational interoperability will sky rocketed.

##### Recommendation 4

In the context of a scaled up infrastructure, nodes in the federation will require a number of profiles: domain experts (depending on the research question), data scientists, data managers and system administrators. The Coordination Hub requires, in addition, an IT profile expert in distributed computing.

##### Recommendation 5

Orchestrating the whole distribution in more complex federations will require a stepwise approach (see details in Appendix 2) that smooths down the exchange between the Coordination Hub and the nodes, while deploying an analytical pipeline that is transparent and reproducible at any step.

##### Recommendation 6

In the institutions composing the federation, rating data curation institutions according to their procedures to get data up-to-date and high quality; agreeing on a common data quality framework (see for example in [13]); cataloguing their data sources in a way that is standard (e.g., DCAT [14]); providing information on interoperability standards and reusability; publishing clear procedures to get access to their data.

#### On semantic interoperability

Data requirements within InfAct JA case studies have been intendedly limited and, consequently, the number of data sources and the data types. Thus, getting a common data model has then been rather uncomplicated. A pan European federated infrastructure that is expected to interact with unlimited research questions will require consider multiple data sources and many more data types. Some of them may come from routine collections; for example, administrative or claims data as the ones used in the stroke case study, disease-specific registries, population-based registries, socioeconomic repertoires, electronic and medical health records, data from lab tests, data from imaging tests, etc. Some of them from ad hoc data collections, e.g., DNA sequences, tissues, data from wearables, samples of texts or data from social media.

In addition to the variety of data sources is also the heterogeneity of data in their very nature (at one end, administrative data; at the other end, natural language) but also heterogeneous in the encoding systems. For example the inclusion of new countries in the stroke use case may have required other disease encoding systems, such as NOMESCO [15], OPCS [16], Leistsungkatalog [17] or ACHI [18]. In case of using data from lab tests LOINC [19] should have been mapped out.

##### Recommendation 7

In the context of a scaled up infrastructure, it would be recommendable to map out and catalogue the most prevalent semantic interoperability standards. In that sense, future initiatives should link to standards developers and curators, for example, using SNOMED [20], the ontology of reference terms for medical conditions.

##### Recommendation 8

A pan european federated infrastructure should link with the existing research infrastructures on health data. On the one hand, to learn how they have catalogued the standards of semantic (and syntactic) interoperability. On the other hand, to provide access to their standards to the population health research community that could be interested in data models including that variety of data sources. As a matter of example, standards on biosamples [21] or omics [22].

##### Recommendation 9

It is expected that most of the studies in an expanded version of a federated research infrastructure, the vast majority of research will be observational studies. A major multiparty initiative pursuing a common data model for observational research is OMOP [23]. A close follow up of this initiative is recommendable, even proactively advocating improvements to get the specificities of population health research well represented in the OMOP CDM.

#### On technological interoperability

Although setting up the foundations for the deployment of more complex pipelines the aforementioned technological elements in the JA-InfAct stroke case study had a very modest scope. The eventual expansion of a federated research infrastructure such as the one tested in InfAct would require a technological upgrade considering three elements: the reduction of human interaction in the steps proposed in 4.2.2; the possibility of heavier computational workloads, and designing the architecture to allow full distribution of complex methodologies whose results are comparable to the ones using data pooling in a single repository.

The JA-InfAct federated analysis infrastructure can be considered a step towards more sophisticated solutions. It is a reliable solution for a problem specific scenario, but the foundations may be easily extended to include more analysis pipelines. For example, a generalized version of the infrastructure may support fully distributed statistical algorithms [24, 25] and, in the final term, state-of-the-art federated learning algorithms [26–28], the current cutting-edge analysis approach when leading with huge data sets distributed across multiple locations, without having the possibility of merging them. In addition, the current client-server architecture, which relies on a Coordination Hub that agglutinates a high level of responsibility can be moved to a peer-to-peer architecture, where all partners/Data Hubs can act as peers, having the capacity to coordinate analyses through the infrastructure.

##### Recommendation 10

When it comes to reducing human interaction, a way forward will be developing and implementing a robust deployment protocol to automatically orchestrate the federation set up activities between the Coordination Hub and the different, detailed in the stepwise process represented in appendix 2.

##### Recommendation 11

One of the tasks of the Coordination Hub in an eventual scaled up federated infrastructure should be the assessment of the computational needs of the different research queries. To optimize the infrastructure resources and reduce the management overheads, it is sensible to outsource the computing or storage capabilities instead of having and maintaining high capacities inhouse. A solid European service providers such as, EGI, for computation capacities https://www.egi.eu, or EUDAT, for storage capacities (https://www.eudat.eu/) are primary choices for this purpose.

##### Recommendation 12

In federated infrastructures, when research questions and research methodologies become more demanding the design of distributed analyses becomes paramount. These new and growing requirements should be faced in a future federated infrastructure by providing state-of-the-art federated machine learning algorithms and methods, as well as to provide the elements to easily develop or adapt new analysis techniques.

##### Recommendation 13

In federated infrastructures, when the algorithm deployed is fully distributed, and no individual data is shared between nodes but only analytic aggregated results, differential privacy is guaranteed as long as each node ensures differential privacy inside their own datasets. In those cases where the algorithm requires individual data sharing, differential privacy is usually guaranteed as there is no data linkage between nodes, i.e., there are no personal identifiers or pseudonyms common to several nodes identifying the same person. In any case, it is always a good practice to analyse the datasets’ characteristics, and enforce privacy through additional processes, like data minimisation or k-anonymisation [29].

## Conclusion

To conclude, it is important to note that all the know-how gathered during the development of the JA-InfAct federated analysis infrastructure and some of the recommendations provided are currently being implemented in PHIRI, population health information research infrastructure [https://www.phiri.eu], a practical roll out of DIPOH, distributed infrastructure on population health research, current candidate to get into the ESFRI roadmap. In addition, all this insight is playing a fundamental part of the European Health Research and Innovation Cloud, a cloud for health data exchange between European health research infrastructures and health services, to be designed under the HealthyCloud project [https://healthycloud.eu]. Finally, this knowledge is currently helping to give shape to the future European Health Data Space, the project to regulate the secondary use of health data across Europe, under the framework of the TEHDaS Joint Action [https://tehdas.eu].

## Data Availability

This research builds on a federated research approach where data holders keep the data in their premises (ie, no individual nor aggregated is pooled centrally) but only scripts and aggregated results move. All scripts have been developed on open source solutions, transparent to be audited, and available under Creative Commons Attribution 4.0 International licensing.

## Appendix 1

### Stepwise approach to get organisational interoperability in a federated infrastructure

In the implementation of a research query, we do recommend building semantic interoperability by following these 8 activities - some of them recursive. In the client-server approach, the Coordination Hub has to orchestrate all the activities across nodes, aiming to reduce human interaction as much as possible.

**Step 1** Any member of the user community could broadcast a research question to the federated research infrastructure.

**Step 2** Once a research question (RQ) has been sent out to the federation of nodes, the Coordination hub will start a process to define the key components of the RQ, so to get a detailed operational definition (i.e. unit of analysis, period of study, target population, inclusion and exclusion criteria, etc.)

Once the research question is clear, the promoter of the query will draft the data schema of the use case defining in detail; so, the data schema will require: the entity, the attribute of interest (variable) with a label and a normative description, the encoding system of the variables, the unit and values in which it is stored, the validation rules, whether the variable is required of optional, and finally the data source from which the variable has been extracted. In addition, the data model will specify the mapping of the variables to different encoding systems.

**Step 3** As a continuation a synthetic dataset based on these data requirements will be produced to document the data model and formalize the analytical pipeline (see next steps) in a transparent and auditable way. The idea is to anticipate and program all the critical elements in the pipelines and solve any potential problem before real data are used.

When it comes to the formalization of the data model there are multiple options; for example, the minimum metadata schema provided by the `dataspice` implementation (both in R, and Python) complying with the Schema.org international standard (see the example below).

*Note*: Steps 2 and 3 are recursive and once the data model is finally approved in the federation. The participation of domain experts and data scientists is key at this point.

**Step 4 Data quality assessment.** Using the synthetic dataset and open sources libraries, the Coordination Hub will prepare a quality analysis in those key variables looking for incompleteness, missingness, outlier values, anomalous distribution, flaw associations, etc. There are open source libraries (e.g., ‘*dlookr*’, ‘*DataExplorer*’, etc.) that provide you with standard scripts for data quality evaluation.

**Step 5.** Configuration of a preliminary analytical script according to the objectives of the study. Iteration will be critical which will help to update and improve the scripts. For that purpose, the exchange between domain experts, data scientists and IT is key, particularly if the distribution implies model assemblement.

**Step 6.** Once steps 3 to 5 are over, the Coordination Hub will package the whole pipeline distributing the solution (for example, providing a containerized solution using *Docker* or *Podman*) to the different nodes. They, then, will transform their own data to the common data schema, and will run the different parts - data quality assessment and analyses.

**Step 7** In this step the results of the analysis are collected and if the research question requires meta-analysis it will get back to the Coordination Hub, who will run the meta-analyses. Depending on the type of meta-analysis, there could be the need for some recursive interaction between the Coordination Hub and the nodes.

**Step 8** All the pipeline is published in a repertoire as ZENODO. It is worth to highlight the need having all these pipeline findable, accesibles, interoperable and reusable in the OPENAIRE community (https://www.openaire.eu) At this very moment the federation has to decide the level of access given to the pipeline (https://guidelines.openaire.eu/en/latest/literature/index_guidelines-lit_v3.html).

## Appendix 2

### Glossary

- **Process-mining:** a series of data analysis techniques able to discover, analyze, and improve business processes based on event data.
- **Event log:** a file or database containing the registry of the actions performed in a system.
- **Common Data Model:** abstract model agreed between multiple organisations, used to represent real-world entities based on organise the description of these entities in data items and their relationships.
- **Interoperability:** ability of systems or products to interact and operate with other systems or products, based on the agreement of common interfaces.
- **Docker:** compendium of existing Linux-based technologies able to create isolated execution environments used to guarantee the reproducibility of the executions.
- **Computing Pipeline:** set of computing tools that are executed in a certain order and whose inputs and outputs are also linked in a certain manner so as to solve a specific problem.

## Abbreviations

FRI: federated research infrastructure
EIF: European Interoperability Framework
ICT: Information and Communication Technologies
SQL: Structured Query Language
EHR: Electronic Health Record
PC: Primary care
ER: Emergency room
RHB: Rehabilitation
FAIR: Findable, Accessible, Interoperable and Reusable
GDPR: General Data Protection Regulation
DPO: Data Protection Officers
CDM: Common Data Model
DCAT: Data Catalogue vocabulary
ETL: Extraction, Transformation and Loading
IT: Information Technologies
CT: Computerised tomography
CSV: comma-separated values
UTF: unicode transformation code

## Footnotes

1 https://www.uml.org/
