# Correction to: Coping with interoperability in the development of a federated research infrastructure: achievements, challenges and recommendations from the JA-InfAct

**DOI:** 10.1186/s13690-022-00877-4

**Published:** 2022-04-11

**Authors:** Juan González-García, Francisco Estupiñán-Romero, Carlos Tellería-Orriols, Javier González-Galindo, Luigi Palmieri, Andrea Faragalli, Ivan Pristās, Jakov Vuković, Janis Misinš, Irisa Zile, Enrique Bernal-Delgado, on behalf of the InfAct Joint Action consortium

**Affiliations:** 1 Biocomputing Unit, Institute for Health Sciences in Aragon (IACS), Zaragoza, Spain; 2 Data Sciences for Health Services and Policy Research, Institute for Health Sciences in Aragon (IACS), Zaragoza, Spain; 3 Department of Cardiovascular, Endocrine-metabolic Diseases and Aging, Istituto Superiore di Sanità-ISS, Rome, Italy; 4 Center of Epidemiology and Biostatistics, Polytechnic University of Marche, Ancona, Italy; 5 Institute of Public Health, Zagreb, Croatia; 6 Centre for Disease Prevention and Control, Riga, Latvia

**Correction to: Arch Public Health 79, 221 (2021)**

**https://doi.org/10.1186/s13690-021-00731-z**

Following publication of the original article [1], the authors reported that the sixth author's family name was incorrectly spelled as "Fagaralli".

The correct spelling of the family name "Faragalli" has been provided in this Correction.

The original article [1] has been updated.
